# Binary gene induction and protein expression in individual cells

**DOI:** 10.1186/1742-4682-3-18

**Published:** 2006-04-05

**Authors:** Qiang Zhang, Melvin E Andersen, Rory B Conolly

**Affiliations:** 1 Division of Computational Biology, CIIT Centers for Health Research, Research Triangle Park, NC 27709, USA; 2 National Center for Computational Toxicology, U.S. Environmental Protection Agency, Research Triangle Park, North Carolina 27711, USA

## Abstract

**Background:**

Eukaryotic gene transcription is believed to occur in either a binary or a graded fashion. With binary induction, a transcription activator (TA) regulates the probability with which a gene template is switched from the inactive to the active state without affecting the rate at which RNA molecules are produced from the template. With graded, also called rheostat-like, induction the gene template has continuously varying levels of transcriptional activity, and the TA regulates the rate of RNA production. Support for each of these two mechanisms arises primarily from experimental studies measuring reporter proteins in individual cells, rather than from direct measurement of induction events at the gene template.

**Methods and results:**

In this paper, using a computational model of stochastic gene expression, we have studied the biological and experimental conditions under which a binary induction mode operating at the gene template can give rise to differentially expressed "phenotypes" (i.e., binary, hybrid or graded) at the protein level. We have also investigated whether the choice of reporter genes plays a significant role in determining the observed protein expression patterns in individual cells, given the diverse properties of commonly-used reporter genes. Our simulation confirmed early findings that the lifetimes of active/inactive promoters and half-lives of downstream mRNA/protein products are important determinants of various protein expression patterns, but showed that the induction time and the sensitivity with which the expressed genes are detected are also important experimental variables. Using parameter conditions representative of reporter genes including green fluorescence protein (GFP) and β-galactosidase, we also demonstrated that graded gene expression is more likely to be observed with GFP, a longer-lived protein with low detection sensitivity.

**Conclusion:**

The choice of reporter genes may determine whether protein expression is binary, graded or hybrid, even though gene induction itself operates in an all-or-none fashion.

## Background

Two operational models, binary and graded, have been proposed for the mechanism of eukaryotic gene induction [1,2]. The binary model contends that at a given moment, a promoter, i.e., the regulatory region of a gene, can only assume one of two discrete transcriptional states: active and inactive. Once in the active state, gene transcription proceeds at a relatively constant rate; whereas in the inactive state, no transcription occurs. With this binary mode of action, transcription activators, repressors and *cis*-acting elements would induce/repress gene expression by affecting, essentially, the probability with which a promoter is switched on/off. In contrast to this all-or-none mode of operation, the graded induction model argues that a promoter can have continuously varying levels of transcriptional activity, and transcription factors regulate gene expression by affecting the rate at which RNA is produced from the gene template.

**Figure 1:**
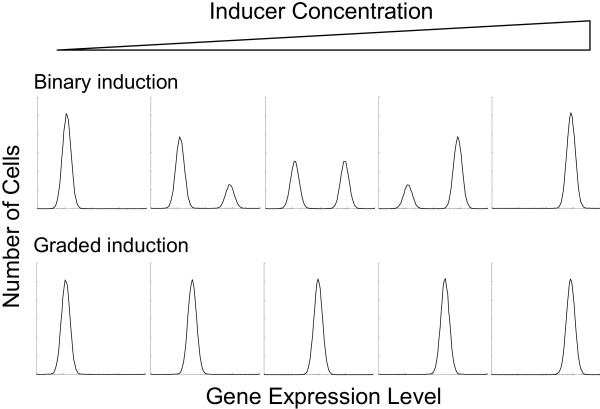
Schematic representation of gene expression histograms for binary and graded modes of gene induction.

To distinguish the two modes of gene induction, fluorescence flow cytometry or microscopy studies are often conducted in individual cells to examine protein expression of either native genes or, in most cases, reporter genes such as green fluorescence protein (GFP) and β-galactosidase (β-gal). Expression data are routinely presented as distribution histograms, in which the x-axis denotes the levels of protein expression and the y-axis represents the number or percentage of cells expressing the reporter protein at different levels (Fig. 1). In a binary induction pattern, two peaks would be seen in the histogram – one representing the cell population expressing the reporter gene, the other representing the population not expressing the gene. Ideally, varying the concentration of transcription inducers would cause changes in the number of cells in each population (i.e., the heights of the peaks), but not the protein levels in the induced cells (i.e., the positions of the peaks along the x-axis). In a graded mode of gene induction, there would only be a single peak in the histogram; varying the concentration of the inducer shifts this single peak along the x-axis.

While observing protein expression in individual cells is informative for gauging the mode of gene induction, caution should be exercised in attempting to infer from protein expression data the manner in which induction events occur at the upstream gene template. In eukaryotic cells where gene promoters may operate in a binary fashion, the half-lives of downstream mRNAs and proteins, relative to the lifespan of the active/inactive promoters, are important determinants for protein expression patterns [3-6]. While early studies using β-gal as a reporter supported a binary mode of gene induction [7-12], increasing numbers of more recent studies using GFP have presented data more indicative of graded mode of induction [13-17]. Given the distinct properties of these two reporter genes with respect to mRNA/protein half-lives [18-25] and detection sensitivity [26-28], the choice of reporter gene may play a significant role in shaping the observed pattern of gene expression. In this paper, using a computational model of stochastic gene expression, which operates in a binary mode at the gene template, we analyzed how the interplay between mRNA and protein half-lives, the lifetime of transcriptionally active promoters, the duration of gene induction, and the sensitivity of protein detection shapes the dynamics and phenotypic patterns of protein expression on a histogram. This evaluation was followed by simulations using parameter conditions compatible with several commonly-used reported genes including GFP, β-gal and luciferase (Luc). We concluded that short mRNA and protein half-lives and induction time, prolonged active state of the promoter, and high sensitivity of detection of reporter proteins favor the appearance of bimodal protein expression; the opposite conditions favor the appearance of graded protein expression. Graded expression is more likely to be observed with GFP, a long-lived reporter protein with low detection sensitivity.

## Results

### Transcription activators (TA) and transcriptionally active/inactive cell populations

**Figure 2:**
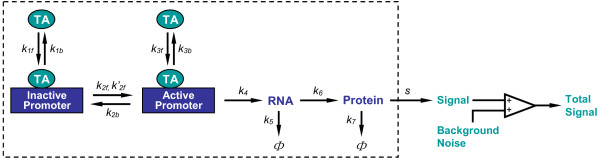
Structure of the stochastic model for binary gene induction. Reactions enclosed in the box were simulated with Gillespie's exact method (see supporting material for reaction details). The reaction volume, i.e., the nucleus volume, was 100 *μ*m^3^. TA: transcription activator. Φ represents RNA and protein degradation. Simulations started with 1 copy of inactive promoter, 0 ~ 512 copies of TA (equivalent to 0 ~ 8 nM), and 0 copies of all other molecule species. *k*_*i*_, *k*_*if *_and *k*_*ib*_, for *i *= 1, 2, ..., 7, are stochastic reaction constants (*k*_2*f *_and *k'*_2*f *_are the TA-dependent and TA-independent activation rates of the promoter, respectively; *k*_5 _= In 2/, *k*_7 _= In 2/, where  and  are RNA and protein half-lives, respectively). *s *is protein detection sensitivity. Unless otherwise indicated, reaction constants *k*_*1f*_, *k*_*1b*_, *k*_*2f*_, *k'*_*2f*_, *k*_*3f*_, *k*_*3b*_, *k*_*4 *_and *k*_*6 *_were fixed for all simulations (*k*_*1f*_, *k*_*3f *_= 1.12 × 10^-4^; *k*_*1b*_, *k*_*3b *_= 1.48 × 10^-2^; *k*_*2f *_= 1.67 × 10^-4^; *k'*_*2f *_= 1 × 10^-9^; *k*_*2b *_= 3.33 × 10^-5^; *k*_*4 *_= 5.56 × 10^-3^; *k*_*6 *_= 4.17 × 10^-3^; unit = s^-1^). Background noise followed normal distribution *N*(10, 3^2^) excluding values less than 1.

In the binary gene induction model (Fig. 2, see Methods for details), the inactive and active promoters represent the transcriptionally activated (on) and repressed (off) states of the gene, respectively. In the absence of TA, most cells in a population are transcriptionally silent owing to the low probability of the promoter switching from the inactive to the active state (*P*_*off*→*on *_= *k'*_2*f*_*δt*). This probability increases after a TA molecule is bound to the promoter, and its average value in the next infinitesimal time interval *δt *can be expressed as:

*P*_*off*→*on *_= *aδt*,     (1)

where 

On an individual gene template basis, 1/*a *determines the average lifetime of the template/promoter remaining inactive prior to being switched on. On a population basis, ln2/*a *relates to the time from the onset of induction to the point where half of the cell population has responded by switching the gene template to active state at least once, while the other half has not responded. The length of time a gene template will remain in the active state before switching back to the inactive state depends on the probability

*P*_*on*→*off *_= *k*_2*b*_*δt*.     (2)

**Figure 3:**
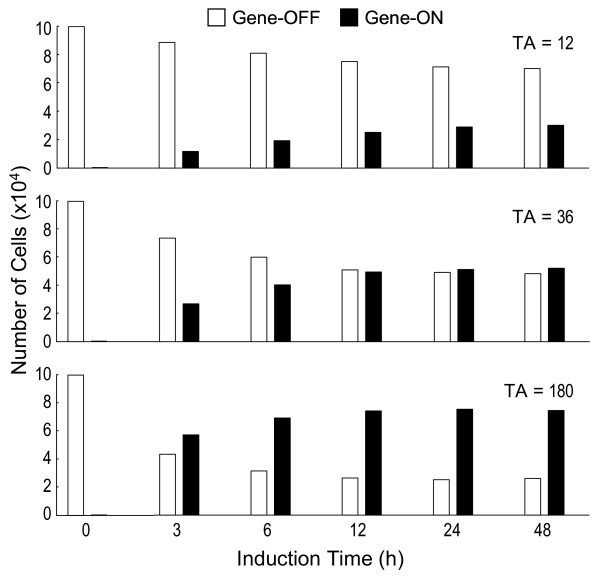
Number of cells that are transcriptionally active (gene-on) and inactive (gene-off) at different induction time points under various TA concentrations.



Conceivably, the population of cells that are transcriptionally active will increase from the onset of induction, whereas those that are transcriptionally inactive will diminish over time (Fig. 3). Eventually a steady state is reached; thereafter the ratio of the two populations remains unchanged. The ratio at the steady state is defined by

The time required to reach half of the steady-state ratio from the onset of induction is



According to Equations (3) and (4), high concentrations of TA cause the steady state to be reached earlier with more cells engaged in the transcriptionally active state (Fig. 3). Although at the population level the steady state will be maintained as long as the inducing condition remains unchanged, the gene template continues to transit between the active and inactive state in individual cells.

### Half-life and protein expression histogram

**Figure 4:**
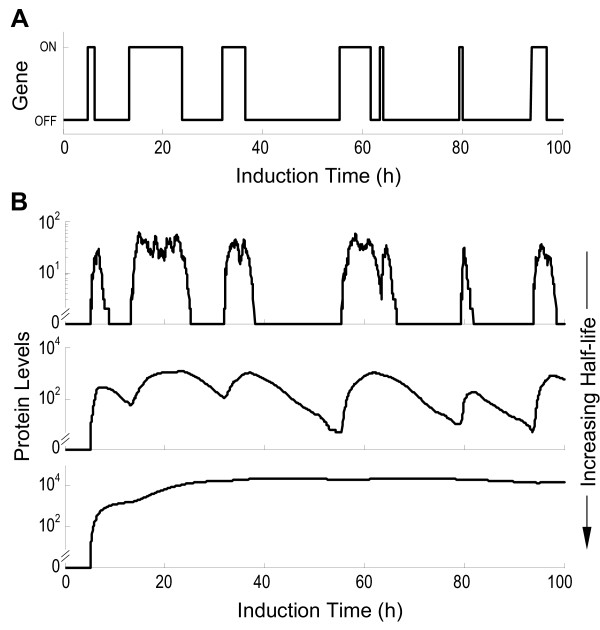
Effect of mRNA and protein half-lives on the dynamics of protein expression. (A) Binary switching of a gene template between the active (on) and inactive (off) state under inducing condition of [TA] = 32. (B) Levels of protein expression in response to the gene template activity in (A), simulated with different RNA and protein half-lives. Top:  = 10 min,  = 20 min; Middle:  = 1 h,  = 2 h; Bottom:  = 8 h,  = 16 h.

The transcriptional status of a gene template is often monitored indirectly by measuring the final protein product. Intuitively, to reflect the transcriptional state of the gene template faithfully (Fig. 4A), the half-lives of both the mRNA and the protein ought to be sufficiently short relative to the lifetimes of the active and inactive promoters. With very short half-lives, protein expression followed gene events closely – the protein level was high when the gene was transcriptionally active and low when it was inactive (Fig. 4B). This tight coupling makes possible a timely monitoring of the ongoing, and even transient, transcriptional event at the gene template, using the protein as a surrogate. In comparison, as mRNA and protein half-lives increased, protein expression levels were less likely to reflect the gene switching fully because the mRNA and/or protein did not disappear quickly. After a few gene on/off cycles, the protein expression level was uncoupled from the actual transcriptional status at the gene template, and was only indicative of the cumulative history of gene on/off events (Fig. 4B).

**Figure 5:**
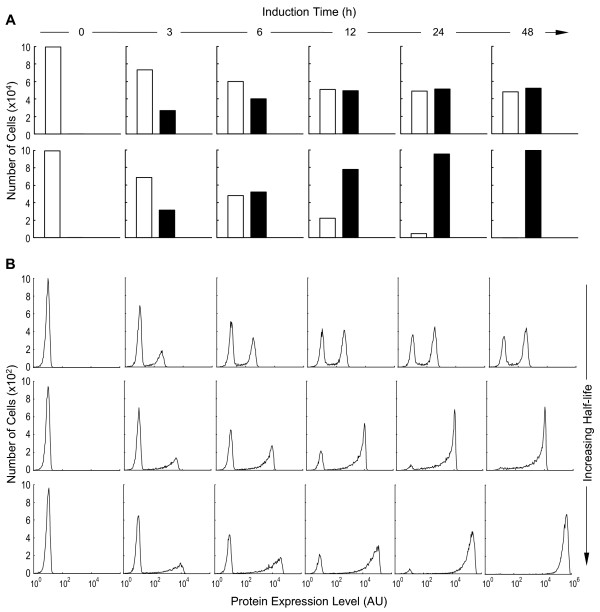
Effect of mRNA and protein half-lives and induction time on the appearance of protein expression histograms. (A) Top: number of cells that are transcriptionally active (blank bar) and inactive (solid bar) at different induction time points under inducing condition of [TA] = 36; Bottom: number of cells that have yet to be induced (blank bar) and those that have been induced (solid bar). (B) Corresponding protein expression histograms (B shares the same time line with A), simulated with different RNA and protein half-lives. Top:  = 10 min,  = 20 min; Middle:  = 1 h,  = 2 h; Bottom:  = 8 h,  = 16 h. AU: arbitrary unit.

On a distribution histogram of protein expression, dual peaks appeared irrespective of the mRNA and protein half-lives (Fig. 5B). As induction time increased, the height of the left peak (representing the number of cells that had either no protein expression or low level expression) decreased, and that of the right peak (representing the number of cells that expressed high levels of the protein) increased, indicating that more cells were recruited to engage in active transcription. With very short mRNA and protein half-lives, a steady-state phase was quickly reached where the ratio of the two peak heights remained unchanged for the rest of the induction time (Fig. 5B, top). This temporal evolution of the two peaks closely resembled the ratio changes between the transcriptionally active and inactive populations (Fig. 5A, top). With increased mRNA and protein half-lives, although the right and left peaks of the histograms still accurately reflected the active/inactive population ratios at the early stage of induction (Fig. 5B, middle and bottom, 3 and 6 h induction time), this resemblance was disrupted as induction continued. Cells in the right peak began to over-represent the transcriptionally active population, and those in the left peak to under-represent the inactive population. This misrepresentation of the actual transcriptional status of the gene in a cell population by the protein expression histogram was noticeable even when the mRNA and protein half-lives were as short as 1 and 2 h respectively (Fig. 5B, middle), the lower end of the half-life ranges in eukaryotic cells [29-32]. Evidently, at the early stage of induction, most of the cells are still in the transcriptionally inactive state and no protein is synthesized; they constitute the left-peak population in the histogram. As soon as the gene template in a cell is switched on, protein synthesis is initiated, and sufficient protein accumulation will move the cell from the left peak to the right peak. Subsequent turning-off of gene transcription in the same cell is not associated with immediate disappearance of the protein owing to the long half-lives, so the cell will remain in the right peak for an extended period until the protein is significantly degraded. In consequence, situations arise in which not all cells in the right peak are actively engaged in transcription despite their high protein levels. It is also conceivable that, given a sufficiently prolonged induction time, which depends on *P*_*off*→*on*_, nearly all cells in the whole population would eventually respond with their gene templates switched on at least once. These cells will join the right peak, making the left peak disappear. Hence with longer mRNA and protein half-lives, the right and left peaks in the histogram fail to mirror cell populations that are transcriptionally active and inactive at the moment of observation. Rather, the two peaks more accurately represent the history of the response (compare Fig. 5A, bottom and Fig. 5B, bottom).

Besides influencing the temporal evolution of peak heights, mRNA and protein half-lives also affected other aspects of the histogram. With longer half-lives, the horizontal position of the right peak shifted progressively to the right as the induction time increased (Fig. 5B). This shift, reflecting increases in the average amount of protein in responsive cells, is explained by protein accumulation over time before a steady state is reached. It takes about five half-lives of either mRNA or protein, whichever is longer, to reach the steady state. Half-lives also affected the shapes of the peaks. With longer half-lives, the right peak, especially at early induction times, was broad and biased towards high protein expression levels with a trailing left tail. This heterogeneity in protein expression, as represented by the broadened geometry, simply reflects the fact that the cells turned gene templates into the active state at different times through the induction period, owing to the stochastic nature of binary switching. Since more cells turned transcriptionally active at the early stage of induction than at the late stage, and since earlier activation of transcription affords a longer time for the protein to accumulate to high levels, the peak on the right was asymmetrically biased. Nevertheless, as induction time increased, this heterogeneity in protein expression diminished considerably because the protein in most cells approached a similar, and eventually steady state, level. Among the three pairs of mRNA and protein half-lives used for simulation (Fig. 4 and 5), 8 h for mRNA and 16 h for protein are close to the respective mean mRNA and protein half-lives in eukaryotic cells [29-32]. Unless otherwise specified, this pair of half-lives was used for subsequent simulations.

### Lifetime of active promoter and induction time

**Figure 6:**
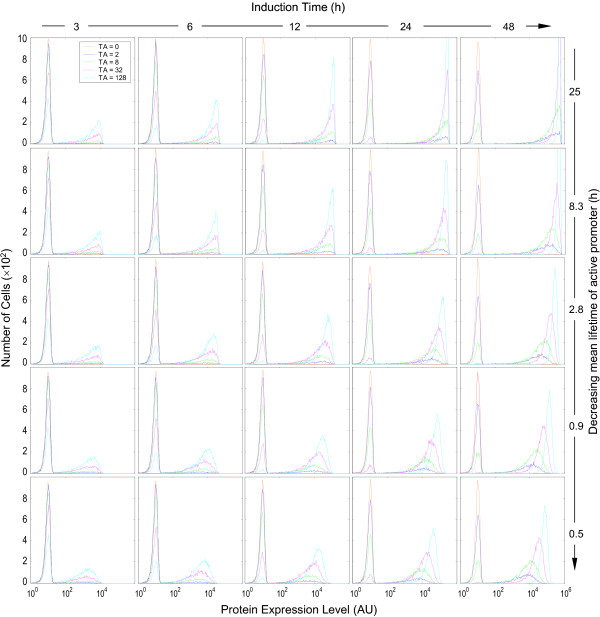
Effect of the mean lifetime of active promoter (1/*k*_*2b*_) and induction time on the appearance of protein expression histograms. Colors coding for TA concentrations are indicated in the top left-most histogram. Values of relevant parameters (s^-1^): *k*_*2b *_= 1.11 × 10^-5 ^~ 60.0 × 10^-5^;  = 8 h;  = 16 h.

Early computational studies indicated that the half-lives of the transcription/translation products, relative to the average lifetimes of the active and inactive promoters, are important factors determining whether the protein expression appears binary or graded [3-6]. A longer promoter lifetime appears to be associated with a binary response, while a shorter one tends to produce a graded response. Our simulation results were consistent with this conclusion. As indicated in Fig. 6, pure binary response patterns were observed with long active promoter lifetimes – increases in inducer concentrations caused lowering of the left peak and heightening of the right peak, with no or little horizontal peak-shifting (top panels). With decreases in the active promoter lifetime the histogram presented a semi-binary and semi-graded appearance (hybrid) – in addition to increases in the height of the right peak, higher TA concentrations also caused rightward shifting and narrowing of the right peak (Fig. 6, bottom panels). Importantly, a complicating factor affecting the binary vs. graded appearance is the induction time, an experimental variable that can range widely. A long active promoter lifetime gave rise to binary protein expression almost independently of the duration of induction. With short-lived active promoters, the appearance of the histograms was also dependent on how long the cells were exposed to the inducers. A very short induction time (3 – 6 h in this case) was marked predominantly by binary responses, while prolonged induction caused separation of the right peaks along the x-axis, resulting in hybrid responses. When the induction time is comparable to the lifetime of the active promoter, gene templates may become active only once, so that the protein level in individual cells is primarily determined by factors (mRNA level, protein half-life, etc.) other than TA concentrations. When the induction time is significantly longer than the lifetime of the active promoter, the gene template may go through several active/inactive cycles within the induction period. Thus, the mean protein level at the end of induction would be determined not only by its half-life, but also by the number of active promoter states experienced, which is proportional to *R*_*on*/*off *_as defined in Equation (3). Evidently, higher TA concentrations are associated with increased *R*_*on*/*off *_thus more active promoter states, leading to higher mean protein levels and rightward shifting of the right peak. As presented below, this horizontal migration of the right peak in response to increasing TA concentrations acts as one of the factors contributing to the appearance of graded protein expression.

### Detection sensitivity

**Figure 7:**
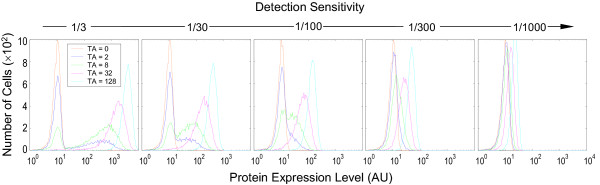
Effect of protein detection sensitivity on the appearance of protein expression histograms. Detection sensitivity is defined as the inverse of the number of protein molecules required to produce a signal intensity equal to the mean background noise signal. Values of relevant parameters (s^-1^): *k*_*2b *_= 3 × 10^-4^;  = 8 h;  = 16 h; induction time = 48 h.

Ideally, monitoring gene transcriptional activity via measuring protein products requires a method sensitive enough to detect relatively few protein molecules efficiently. In practice, the detection sensitivity varies greatly among different reporter genes. Enzyme markers such as β-gal afford very high sensitivities [28], whereas tens of thousands GFP molecules are usually required to make the fluorescence signal discernible over the background noise [26,27]. A potential consequence of using low-sensitivity markers is that at the time of measurement, especially at an early stage of induction, protein molecules may not have accumulated to detectable levels. In a histogram, these cells, although actively transcribing or having transcribed the gene, will remain in the left-peak population. Should this occur, the left peak will over-represent cells that have yet to respond to the inducer. Besides the potential inflation of the left peak, lower sensitivity also causes leftward shifting of the right peak because the signal is reduced (Fig. 7). As the sensitivity is decreased further, the right and left peaks first overlap at some points, then merge into a single, albeit initially broad peak. This effect, when combined with the hybrid response produced when the lifetime of the active promoter is short and induction is long, can give rise to a more complete appearance of graded protein expression (Fig. 7). Thus, the interplay between factors including mRNA and protein half-lives, lifetime of active promoter, induction time and detection sensitivity, coordinately shapes the appearance of protein expression histograms. Appropriate combinations of parameter values for these factors provide the potential to observe binary, hybrid and graded protein expression.

### Protein expression histograms of β-gal, Luc and GFP

**Figure 8:**
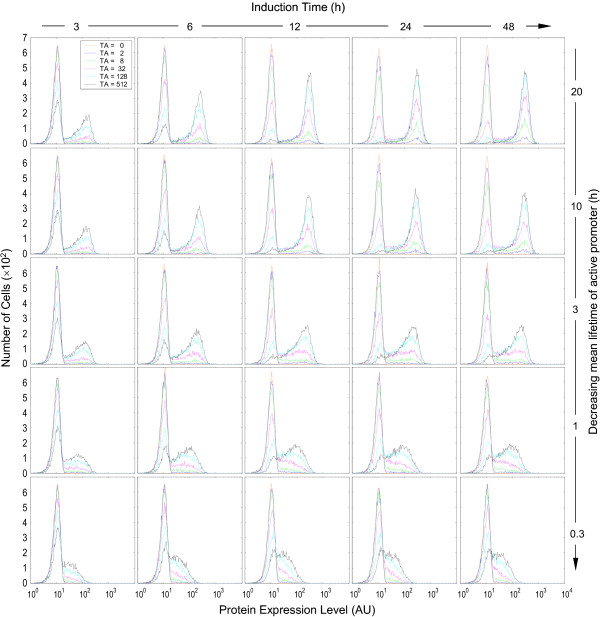
Protein expression histograms obtained with parameter conditions compatible with reporter gene β-gal. Values of relevant parameters (s^-1^): *k*_*2f *_= 1 × 10^-4^; *k*_*2b *_= 1.38 × 10^-5 ^~ 92.6 × 10^-5^; *k*_*4 *_= *N*(5.56 × 10^-3^, 6.94 × 10^-7^); *k*_*5 *_= *N*(1.93 × 10^-4^, 8.34 × 10^-10^) (mean  = 1 h); *k*_*6 *_= *N*(4.17 × 10^-3^, 3.91 × 10^-7^); *k*_*7 *_= *N*(1.93 × 10^-4^, 8.34 × 10^-10^) (mean  = 1 h). Detection sensitivity s = 1/20.

**Figure 9:**
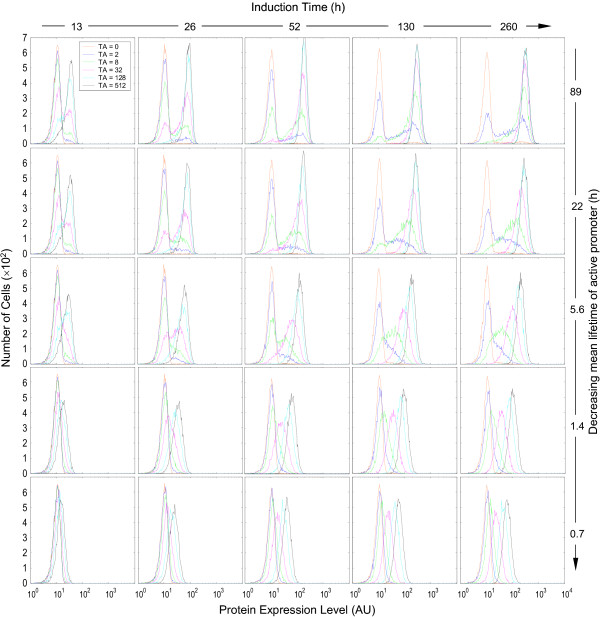
Protein expression histograms obtained with parameter conditions compatible with reporter gene GFP. Values of relevant parameters (s^-1^): *k*_*2f *_= 1 × 10^-4^; *k*_*2b *_= 0.31 × 10^-5 ^~ 40.0 × 10^-5^; *k*_*4 *_= *N*(5.56 × 10^-3^, 6.94 × 10^-7^); *k*_*5 *_= *N*(1.93 × 10^-5^, 8.34 × 10^-12^) (mean  = 10 h); *k*_*6 *_= *N*(4.17 × 10^-3^, 3.91 × 10^-7^); *k*_*7 *_= *N*(7.41 × 10^-6^, 1.23 × 10^-12^) (mean  = 26 h). Detection sensitivity s = 1/5000.

In examining different mode of gene induction, several reporter genes have been used. To investigate how the choice of reporter gene may affect the expression pattern, we simulated gene induction with parameter conditions compatible with the commonly-used reporter genes β-gal, Luc and GFP. With β-gal (Fig. 8) and Luc (supporting material, Fig. S1), binary two-peaked histogram patterns were consistently observed – higher TA concentrations were associated with higher right peaks and lower left peaks. However, under conditions of short-lived active promoter (large *k*_*2b *_values) and long induction time, the strict binary pattern became less apparent – TA at different concentrations caused not only changes in peak heights, but also shifting of the right peak. In consequence, the histograms exhibited hybrid responses of various degrees. Regardless of this hybrid appearance, under no conditions were pure graded responses observed, as two populations of cells could almost always be identified in each histogram. As with β-gal, GFP histograms evolved from a binary to a hybrid appearance as the lifetime of active promoter decreased. But when the mean lifetime of active promoter dropped below 3 h, graded response patterns began to emerge; only a single peak was present, which migrated to the right with increasing TA concentrations (Fig. 9).

The long half-life of traditional GFP [21] makes it difficult to monitor dynamic changes of transient gene transcription. To circumvent this problem, several research laboratories have recently developed destabilized GFPs with significantly shorter half-lives [33,34]. Although these GFPs are expected to provide better time resolution for gene transcription events, our simulation revealed that unless the mRNA half-life is also significantly reduced and detection sensitivity enhanced, graded responses can still be observed with destabilized GFPs under certain conditions, though with lower magnitude (supporting material, Fig. S2).

## Discussion

It has been hypothesized that gene induction occurs in either a binary, on/off or a graded, rheostat-like manner in response to varying inducer concentrations [1,2,35]. Apparent support for each of these two views has come primarily from experimental studies measuring reporter proteins in individual cells, rather than from direct monitoring of molecular events occurring at the gene template in the nucleus [7-17]. With this indirect approach, it is difficult to determine whether different transcriptional responses observed at the protein level (binary, graded or hybrid) are accurate reflections of the respective modes of induction operating at the gene template; rather, these observations may represent differentially expressed "phenotypes" of a single mode of induction operating at different biological and experimental conditions for different gene products.

In the present study using a stochastic computational model, we demonstrated that binary induction at the gene template is capable of producing a wide variety of protein expression patterns. While confirming the importance of lifetimes of active/inactive promoters and of downstream transcription/translation products for determining the appearance of protein expression patterns [3-6], we found that the duration of gene induction and the sensitivity of reporter gene detection are also relevant experimental variables. Specifically, short mRNA and protein half-lives and induction time, prolonged active promoter lifetime and high detection sensitivity favor the appearance of binary protein expression. The reverse conditions favor the appearance of graded protein expression. Binary responses observed with commonly-used reporter genes indicate populations of cells that have or have not responded to the inducing conditions, rather than populations that are engaged or not engaged in active transcription, at the time of examination. Among these reporter genes, GFP has longer mRNA and protein half-lives than β-gal and Luc [18-25]. Equally importantly, since β-gal and Luc are enzyme reporters, the protein signal can be amplified through enzymatic catalysis. In the case of β-gal, as few as five molecules can produce a significant signal for detection [28]. In contrast, tens of thousands GFP molecules are usually required for reliable detection above the background auto-fluorescence [26,27]. The present study demonstrated that β-gal and Luc failed to present complete graded response patterns, whereas such patterns could be readily observed with GFP, which is longer-lived and has much lower detection sensitivity. Given these results, it is less puzzling to note that evidence supporting the binary mode of gene expression first came from early studies using β-gal as the reporter [7-12], whereas the graded mode was observed only when GFP began to be widely used [13-17]. It would be intriguing to see whether those graded responses observed with GFP can be replaced with binary ones if β-gal is used as the reporter protein. In addition to the binary and graded protein expression patterns, the gene induction model also captured an array of intermediate responses – both the percentage of cells expressing the protein and the level of protein in these cells were increased with higher concentrations of the inducer. Similar hybrid responses have been observed in studies using GFP as a reporter [15,16].

In the process of gene induction, the chromatin undergoes decondensation and recondensation, corresponding to the transition between the inactive and active promoter states in the model. This transition, compared with the rapid exchange between transcription factors and promoters, occurs much less frequently, and the promoter may remain active or inactive for hours or longer before changing its state [36]. For an mRNA/protein pair with half-lives considerably shorter than the average lifetime of the active promoter, this would invariably give a binary appearance on protein expression histograms independently of the length of induction time; and the ratio between the right and left peaks increases as induction time lengthens until promoter transition in the entire population reaches a steady state. For an mRNA/protein pair with half-lives comparable to or longer than the lifetime of the active promoter, the induction time starts to affect the binary vs. graded appearance of the protein histograms. For a short-period exposure to an inducer, promoters in most cells either remain inactive or become active only once, giving rise to a binary appearance with protein levels in most cells at a non-steady state. As induction time lengthens, cells can experience two or more inactive/active promoter cycles. The number of cycles increases with higher inducer concentrations, as discussed in the Results section. More promoter cycles within an induction period allow the protein to accumulate to higher levels until a steady state, though a fluctuating one, is reached. Thus, prolonged induction enhances the separation of different steady-state protein expression levels, and increases the likelihood of observing a graded appearance on a protein histogram. As induction time is a controllable experimental variable, it can vary widely relative to the time required for cells to reach the steady state for either promoter transition or protein accumulation. Therefore, when characterizing the mode of gene induction from protein expression data, the length of induction time may need to be taken into consideration.

A key step in the stochastic binary model is the reversible transition of the promoter between the inactive and active states [37,38]. This transition, probably involving multiple biological steps, is governed kinetically by the switching probabilities dictated by Equations (1) and (2). Many nuclear factors can be potential modulators of the switching kinetics. For example, *cis*-acting enhancers can increase the percentage of gene-expressing cells [12] presumably by augmenting *k'*_2*f *_or *k*_2*f *_in Equation (1). Co-activators possessing HAT activity, such as steroid receptor coactivators (SRC), P300/CBP and PCAF, can also play various roles in augmenting *k*_2*f*_. They are usually recruited to the promoter after nuclear receptors bind to the response elements, facilitating the transition from a transcriptionally repressed to a transcriptionally active promoter by diminishing the local interactions between DNA and histone [39]. In accordance with this *k*_2*f *_-augmenting role, co-activator SRC-1 was shown to increase the percentage of responsive cells in glucocorticoid receptor-mediated gene expression [40]. In contrast to the up-regulating controls, negative regulators such as transcriptional repressors can attenuate the positive contribution of TA to *P*_*off*→*on *_by competing for the promoter binding sites, or by binding TA to block its DNA-binding or activational domains. Interestingly, in two studies reporting that the same promoters were capable of producing binary and graded expression responses under different experimental manipulations, the binary responses were observed under more transcriptionally repressed conditions [14,15]. These repressing conditions may slow the transition between the inactive and active promoters, increasing the chance of observing binary responses [3-6]. In Equation (2), *P*_*on*→*off *_determines the length of time a promoter will remain in the active state before it transits to the inactive state. Transcription co-repressors such as NCoR and SMRT facilitate the transition by recruiting HDACs, which assist in chromatin condensation through histone deacetylation [41]. These factors add to the probability *P*_*on*→*off*_, reducing the active promoter lifetime.

At least two distinct mechanisms can give rise to a binary appearance of protein expression. One is stochastic gene activation at the promoter level as described in the present study; the other is binary activation of TA in response to upstream signaling. With stochastic gene activation, the all-or-none response of protein expression lies in the promoter switching between the structurally relaxed (transcriptionally active) and compact (transcriptionally inactive) states, with some probabilities governing the kinetics of the occurrence. This stochastic switching at the gene template in eukaryotic cells has been experimentally demonstrated in recent studies and it contributes greatly to the heterogeneity in gene expression among individual cells [37,38]. When gene switching in a single cell is a random event, successful occurrence of the switching will depend on appropriate stochastic interplay between relevant transcription factors and the promoter. According to this probabilistic view, divergence of gene expression in a population of cells does not rely on extrinsic cell-to-cell variations, and can occur even when the population is otherwise homogeneous.

An alternative explanation for binary protein expression considers that the all-or-none response does not originate at the level of the gene template; rather, it stems from binary activation of the transcription activator [10,14]. With this mechanism, an all-or-none type of ultrasensitive molecular circuit with threshold often exists between the inducer and TA, while the gene template itself can transcribe at continuously varying levels. Graded inducer concentrations are converted to an all-or-none type of response at the TA level, leading to downstream binary induction of gene expression in the cell. Switch-like ultrasensitivity can arise from modular circuits such as zero-order reactions, positive feedbacks or cooperative molecular interactions [42-44]. For continuous changes in the ratio of the two diverging cell populations to be observable in response to varying inducer concentrations, cells must vary broadly in either the threshold value or concentrations/activities of key intermediate signaling molecules driving the switch circuit, regardless of the origin of ultrasensitivity [35].

To distinguish the two sources of binary gene expression, one approach is to measure the level of active transcription factors in individual cells. In one study showing that activation of transcription factor p53 followed an uniform graded distribution in response to genotoxic chemical stressors, the downstream gene expression driven by p53 was found to be either binary or graded, depending on the type of promoters used and on the cell line [17]. In Jurkat T cells, where the cell surface marker CD69 exhibited a binary expression pattern in response to PMA, JNK protein, a downstream kinase responsible for CD69 induction, appeared to have a similar binary distribution [45]. These studies demonstrated that binary gene expression can arise either at the gene template or at the level of the transcription factors and further upstream. Further supporting the stochastic over the threshold mechanism, many studies have shown that in a cell population displaying binary gene expression, each of the sorted low- and high-expressing subpopulations subsequently exhibited similar divergence in gene expression after re-exposure to the same inducers [10,11,17,40]. Were the two subpopulations of cells inherently different – for instance, in the threshold value in an ultrasensitive circuit – their responses to a second induction would probably have remained unchanged, i.e., either low or high. An additional line of evidence supporting the stochastic mechanism is the observation that a longer induction time is often associated with more induced cells [9,10,17], which suggests that whether or not gene expression is induced is simply a matter of time. Had the binary response been governed solely by a switch-like circuit upstream of the gene template, all the induced cells would have all responded at a similar time rather than spread over a much broader time window. Taken together, these data suggest that although switching circuitry with threshold is a potential source of binary gene expression, it is unlikely to be the sole mechanism underlying dichotomous gene induction. The choice to transcribe or not could be probabilistic, made at the level of the gene promoter.

In conclusion, the stochastic model of gene expression demonstrates that a simple binary mode of gene induction can give rise to multiple protein expression patterns – binary, graded and hybrid. The appearance of various response patterns depends on the lifetime of transcriptionally active promoters, half-lives of mRNAs and proteins, duration of gene induction, and sensitivity with which the expressed proteins are detected. To monitor gene induction events accurately, reporters of short mRNA and protein half-lives and high detection sensitivity are desirable.

## Methods

### Model structure

In eukaryotic cells, the protein-encoding genes are believed to be expressed as follows. A transcription activator (TA), in its active form, binds a specific response element in the promoter region of a target gene. Once associated with the promoter, the TA can acts as a platform to recruit to the local promoter region a battery of transcriptional co-regulators such as those possessing histone acetyltransferase (HAT) and histone methyltransferase (HMT) activities and the ATP-dependent chromatin remodeling complex SWI/SNF [39]. Aggregation of these factors at the promoter loosens the structure of the local chromatin, which is usually packed in the condensed form of nucleosomes. The relaxed chromatin structure greatly increases the accessibility of basal transcription factors and RNA polymerase II to the promoter, and correct assembly of these components at the transcription initiation site launches transcription. Nuclear enzymes, including histone deacetylase (HDAC), are also at work to limit gene transcription by reconverting the relaxed chromatin to the compact, transcriptionally repressed form [46]. Multiple rounds of transcription initiation could occur while the promoter is in the active state before it shuts off.

We used a stochastic gene induction model similar to those used by others [3,4,6,37,38]. The binary mode of gene induction was largely implemented through stochastic transition between the transcriptionally active and inactive states of the promoter, which correspond to the relaxed and compact structures, respectively (Fig. 2). Once the promoter is active, transcription proceeds at a pre-determined rate; once the promoter is inactive, transcription ceases. Our model, however, incorporated the recent hit-and-run concept as far as promoter activation is concerned [47,48]. Classically, interactions between the TA and promoter are viewed as a static process – after the TA binds the promoter it remains there for continued gene activation. Recent photobleaching studies performed on single cells have revealed that the TA interacts with promoters in a remarkably dynamic manner – it exchanges on and off the promoter rapidly in the order of seconds to minutes [49-52]. During its transient residence on the promoter, the TA increases the probability of the promoter switching from the inactive to the active state. Maintenance of the active state, however, does not require continued occupancy of the promoter by the TA. In the absence of the TA, the transition from the inactive to the active state, representing macroscopic basal expression, may occur, but with extremely low probability. In the model, reverting from the active to the inactive promoter is regarded as a TA-independent process, and occurs with a fixed probability. In contrast to the rapid association and dissociation between the TA and promoter, the transition between the active and inactive states occurs on a much slower time scale, in the order of hours, as suggested by studies on chromatin remodeling [36]. We assume that the protein product in a cell produces fluorescence/luminescence, the intensity of which is proportional to the amount of the protein. The total signal gathered from a cell is the sum of that contributed by the protein and background noise.

### Model parameters

The stochastic reactions and the values of the reaction parameters are listed in Table S1~S3 in the supporting material, where references and rationale for the choice of parameter values are also given. Each cell is assumed to contain only one copy of the gene template. Unless otherwise indicated, the reaction constants *k*_*1f*_, *k*_*1b*_, *k*_*2f*_, *k'*_*2f*_, *k*_*3f*_, *k*_*3b*_, *k*_*4*_, and *k*_*6 *_were fixed for all simulations (Fig. 2, legend). Wherever cell-to-cell variability was considered, the reaction constants for RNA synthesis (*k*_*4*_) and degradation (*k*_*5*_), and for protein synthesis (*k*_*6*_) and degradation (*k*_*7*_), were drawn from respective normal distributions of *N*(*μ, σ*^2^), where *μ *is the mean and *σ*^2 ^is the variance. Detection sensitivity is defined as the inverse of the number of protein molecules required to produce a signal intensity equal to the mean background noise. The background noise is assumed to follow a normal distribution in a cell population. Each histogram of protein expression distribution was obtained by running the simulation 10^4 ^times and with a bin size of 200.

### Modeling tools

The stochastic simulation used Gillespie's exact method [53] and was implemented in BioNetS developed by Adalsteinsson et al. [54] and MatLab (The MathWorks, Inc., Natick, MA). The model in the BioNets format and MatLab code can be requested from Dr. Zhang at qzhang@ciit.org.

## Competing interests

The author(s) declare that they have no competing interests.

## Supplementary Material

Additional File 1 This file contains parameter values for the model presented in the main text, and some additional simulation results. The file is in pdf format. Click here for file
