# Supplementary material for: Binary gene induction and protein expression in individual cells
Source: Theor Biol Med Model. 2006 Apr 5;3:18. doi: 10.1186/1742-4682-3-18 (PMC1488830; doi:10.1186/1742-4682-3-18)
Supplement: Additional File 1 — This file contains parameter values for the model presented in the main text, and some additional simulation results. The file is in pdf format. [file 1742-4682-3-18-S1.doc]

**Supporting Material**

Binary gene induction and protein expression in individual cells

Qiang Zhang, Melvin E. Andersen, and Rory B. Conolly

**Table S1.** Stochastic reactions and reaction constants.

| **Reaction #** | **Stochastic Reactions** | | |
| --- | --- | --- | --- |
| (1) | TA + DNA_i_ |  | TA_DNA_i_ |
| (2) | TA_DNA_i_ |  | TA_DNA_a_ |
|  | DNA_i_ |  | DNA_a_ |
| (3) | TA + DNA_a_ |  | TA_DNA_a_ |
| (4) |  | | RNA |
| (5) | RNA |  | *Φ* |
| (6) |  | | PROTEIN |
| (7) | PROTEIN |  | *Φ* |

Abbreviations: TA: transcription activator, DNA_i_: inactive promoter, DNA_a_: active promoter; *Φ*: degradation.

**Table S2.** Stochastic reaction constant values

| **Parameter** | **Value (s^-1^)** | **Notes** |
| --- | --- | --- |
| *k_1f_* | 1.12×10^-4^ | These values are derived from binding kinetics measured between estradiol-liganded estrogen receptor dimer and the estrogen response element [1, 2]. With these values, the dissociation constant K_d_ is 2 nM, and the mean TA residence time on the promoter is 67 s. This residence time is compatible with the rapid exchange (seconds to minutes) observed between a variety of transcription factors and response elements [3-5] |
| *k_1b_* | 1.48×10^-2^ |
| *k_2f_* | 1.67×10^-4^ | Unless otherwise specified, this value is chosen so that under intermediate level of induction (TA = 36), most cells would have switched the gene on at least once by 48 h. For β-gal, Luc and GFP simulation, this value was set at 1×10^-4^. |
| *k'_2f_* | 1.0×10^-9^ | This value is set low so that in the absence of TA, basically no gene template is switched on within the maximum induction time period. |
| *k_2b_* | 0.31×10^-5^  ~  92.6×10^-5^ | The inverse of this parameter (1/*k_2b_*) defines the mean lifetime of active promoter. Its value was varied systematically to explore its effect on the mode of protein expression. In simulations where *k_2b_* was not varied, the value was set at 3.33×10^-5^, equivalent to 9 h of lifetime of active promoter. |
| *k_3f_* | 1.12×10^-4^ | The same as *k_1f_* and *k_1b_*, respectively. Since in the model switching from active to inactive promoter is TA-independent, the simulation results are largely insensitive to these two parameters. |
| *k_3b_* | 1.48×10^-2^ |
| *k_4_* | 5.56×10^-3^ | Eukaryotic protein-encoding genes are rarely transcribed by more than one RNA polymerase II at a time [6]. β-gal has the longest coding sequence (~3kb) among the three reporter genes explored in this study. Given an average elongation rate of 2kb/min [7-9], the elongation time for β-gal will be about 1.5 min. The value of *k_4_* is chosen so that on average there is no simultaneous transcription by more than one polymerase on the same gene template, but also the transcription rate is maximized. The value 5.56×10^-3^ (equivalent to 20 RNA molecules produced per h) gives a transcription initiation interval of 3 min, longer than the elongation time for β-gal. Additionally, the simulation results are not sensitive to *k_4_*. |
| *k_5_* | 1.93×10^-4^/$t_{\frac{1}{2}\left(RNA\right)}$ | *k_5_* varies with RNA half-life in h. 1.93×10^-4^ is equivalent to a half-life of 1 h. |
| *k_6_* | 4.17×10^-3^ | 4.17×10^-2^ is equivalent to translation rate of 150 protein molecules per h per mRNA template, an average found in eukaryotic cells [10, 11]. Considering that only a small fraction of primary transcripts reach the cytoplasm as mature mRNA [11], 4.17×10^-2^ is divided by a factor of 10 to account for this reduction. |
| *k_7_* | 1.93×10^-4^/$t_{\frac{1}{2}\left(protein\right)}$ | *k_7_* varies with protein half-life in h. 1.93×10^-4^ is equivalent to a  half-life of 1 h. |

**Table S3.** Parameter values for reporter genes.

| **Reporter** | **Parameter** | **Notes** |
| --- | --- | --- |
| β-gal | $t_{\frac{1}{2}\left(RNA\right)}$ = 1 h | E. coli β-gal mRNA half-life in CV-1 cells ranged from 60 to 75 min [12]. We used 1 h. |
|  | $t_{\frac{1}{2}\left(protein\right)}$= 1 h | Although β-gal was shown to degrade at a half-life of about 20 h or less in certain cell types [13, 14], studies supporting binary gene induction showed that the steady state level of β-gal in individual T cells was achieved in a few hours following induction [15, 16]. This suggested that the half-life of the enzymatic β-gal is less than 1 h in these cells. Therefore 1 h was chosen for the β-gal protein half-life. |
|  | s = 1/20 | As few as 5 molecules are the lower detection limit for β-gal [17]. Since enzymatic β-gal is a tetramer, and tetramization was not modeled in our study, we used 20 (5×4) as the detection limit. |
| Luc | $t_{\frac{1}{2}\left(RNA\right)}$ = 6 h | This half-life value is derived numerically (given that the protein half-life is 3 h) on the basis of a study in which Luc protein expression over time was measured following Luc mRNA delivery to B16-F10, a mouse melanoma cell line [18]. The derived value is the same as Promega (Madison, WI) provided for firefly luciferase mRNA. |
|  | $t_{\frac{1}{2}\left(protein\right)}$= 3 h | The half-life of commonly used firefly Luc in mammalian cells could range from 50 min to 3.68 h depending on variants of Luc and cell types [19-22]. We used 3 h. |
|  | s = 1/50 | No specific information is available for the detection limit of Luc at individual cell level. We assumed the lower limit to be 50 molecules. |
| GFP | $t_{\frac{1}{2}\left(RNA\right)}$ = 10 h | This half-life value is derived numerically (given the protein half-life is 26 h) on the basis of a study in which GFP protein expression over time was measured following GFP mRNA delivery to B16-F10, a mouse melanoma cell line [18]. |
|  | $t_{\frac{1}{2}\left(protein\right)}$= 26 h | Wide-type GFP protein half-life is generally believed to be longer than a day. We used 26 h obtained in mouse LA-9 cells [23, 24] |
|  | Destabilized  $t_{\frac{1}{2}\left(protein\right)}$= 2 h | Several versions of destabilized GFP exist with different half-lives ranging from few to about 10 h [23, 24]. We used 2 h. |
|  | s = 1/5000 | It is believed that at least tens of thousands GFP molecules are usually required for reliable detection above the background auto-fluorescence [25, 26]. We used 5000 as the minimal number of GFP molecules. |

**Figure Legends**

Figure S1. Protein expression histograms obtained with parameter conditions compatible with reporter gene luciferase. Values of relevant parameters (s^-1^): *k_2f_* = 1×10^-4^; *k_2b_* = 1.26×10^-5^ ~ 92.6×10^-5^; *k_4_* = *N*(5.56×10^-3^, 6.94×10^-7^); *k_5_* = *N*(3.21×10^-5^, 2.32×10^-11^) (mean $t_{\frac{1}{2}\left(RNA\right)}$= 6 h); *k_6_* = *N*(4.17×10^-3^, 3.91×10^-7^); *k_7_* = *N*(6.42×10^-5^, 9.27×10^-11^) (mean $t_{\frac{1}{2}\left(protein\right)}$= 3 h). Detection sensitivity s = 1/50.

Figure S2. Protein expression histograms obtained with parameter conditions compatible with destabilized GFP. Values of relevant parameters (s^-1^): *k_2f_* = 1×10^-4^; *k_2b_* = 0.35×10^-5^ ~ 83.3×10^-5^; *k_4_* = *N*(5.56×10^-3^, 6.94×10^-7^); *k_5_* = *N*(1.93×10^-5^, 8.34×10^-12^) (mean $t_{\frac{1}{2}\left(RNA\right)}$= 10 h); *k_6_* = *N*(4.17×10^-3^, 3.91×10^-7^); *k_7_* = *N*(9.63×10^-5^, 2.09×10^-10^) (mean $t_{\frac{1}{2}\left(protein\right)}$= 2 h). Detection sensitivity s = 1/5000.

**Figure S1**

**Figure S2**

**Supporting Material References**

1. Szatkowski Ozers M, Hill JJ, Ervin K, Royer CA, Gorski J: **The dissociation rate of estrogen receptor alpha from the consensus estrogen response element**. *Mol Cell Endocrinol* 2001, **175**(1-2):101-109.

2. Ozers MS, Hill JJ, Ervin K, Wood JR, Nardulli AM, Royer CA, Gorski J: **Equilibrium binding of estrogen receptor with DNA using fluorescence anisotropy**. *J Biol Chem* 1997, **272**(48):30405-30411.

3. Stenoien DL, Nye AC, Mancini MG, Patel K, Dutertre M, O'Malley BW, Smith CL, Belmont AS, Mancini MA: **Ligand-mediated assembly and real-time cellular dynamics of estrogen receptor alpha-coactivator complexes in living cells**. *Mol Cell Biol* 2001, **21**(13):4404-4412.

4. Becker M, Baumann C, John S, Walker DA, Vigneron M, McNally JG, Hager GL: **Dynamic behavior of transcription factors on a natural promoter in living cells**. *EMBO Rep* 2002, **3**(12):1188-1194.

5. McNally JG, Muller WG, Walker D, Wolford R, Hager GL: **The glucocorticoid receptor: rapid exchange with regulatory sites in living cells**. *Science* 2000, **287**(5456):1262-1265.

6. Jackson DA, Iborra FJ, Manders EM, Cook PR: **Numbers and organization of RNA polymerases, nascent transcripts, and transcription units in HeLa nuclei**. *Mol Biol Cell* 1998, **9**(6):1523-1536.

7. Shermoen AW, O'Farrell PH: **Progression of the cell cycle through mitosis leads to abortion of nascent transcripts**. *Cell* 1991, **67**(2):303-310.

8. Femino AM, Fay FS, Fogarty K, Singer RH: **Visualization of single RNA transcripts in situ**. *Science* 1998, **280**(5363):585-590.

9. Tennyson CN, Klamut HJ, Worton RG: **The human dystrophin gene requires 16 hours to be transcribed and is cotranscriptionally spliced**. *Nat Genet* 1995, **9**(2):184-190.

10. Arava Y, Wang Y, Storey JD, Liu CL, Brown PO, Herschlag D: **Genome-wide analysis of mRNA translation profiles in Saccharomyces cerevisiae**. *Proc Natl Acad Sci U S A* 2003, **100**(7):3889-3894.

11. Jackson DA, Pombo A, Iborra F: **The balance sheet for transcription: an analysis of nuclear RNA metabolism in mammalian cells**. *Faseb J* 2000, **14**(2):242-254.

12. Fuerst TR, Moss B: **Structure and stability of mRNA synthesized by vaccinia virus-encoded bacteriophage T7 RNA polymerase in mammalian cells. Importance of the 5' untranslated leader**. *J Mol Biol* 1989, **206**(2):333-348.

13. Bachmair A, Finley D, Varshavsky A: **In vivo half-life of a protein is a function of its amino-terminal residue**. *Science* 1986, **234**(4773):179-186.

14. Smith RL, Geller AI, Escudero KW, Wilcox CL: **Long-term expression in sensory neurons in tissue culture from herpes simplex virus type 1 (HSV-1) promoters in an HSV-1-derived vector**. *J Virol* 1995, **69**(8):4593-4599.

15. Karttunen J, Shastri N: **Measurement of ligand-induced activation in single viable T cells using the lacZ reporter gene**. *Proc Natl Acad Sci U S A* 1991, **88**(9):3972-3976.

16. Fiering S, Northrop JP, Nolan GP, Mattila PS, Crabtree GR, Herzenberg LA: **Single cell assay of a transcription factor reveals a threshold in transcription activated by signals emanating from the T-cell antigen receptor**. *Genes Dev* 1990, **4**(10):1823-1834.

17. Fiering SN, Roederer M, Nolan GP, Micklem DR, Parks DR, Herzenberg LA: **Improved FACS-Gal: flow cytometric analysis and sorting of viable eukaryotic cells expressing reporter gene constructs**. *Cytometry* 1991, **12**(4):291-301.

18. Bettinger T, Carlisle RC, Read ML, Ogris M, Seymour LW: **Peptide-mediated RNA delivery: a novel approach for enhanced transfection of primary and post-mitotic cells**. *Nucleic Acids Res* 2001, **29**(18):3882-3891.

19. Leclerc GM, Boockfor FR, Faught WJ, Frawley LS: **Development of a destabilized firefly luciferase enzyme for measurement of gene expression**. *Biotechniques* 2000, **29**(3):590-591, 594-596, 598 passim.

20. Nunez L, Faught WJ, Frawley LS: **Episodic gonadotropin-releasing hormone gene expression revealed by dynamic monitoring of luciferase reporter activity in single, living neurons**. *Proc Natl Acad Sci U S A* 1998, **95**(16):9648-9653.

21. Thompson JF, Hayes LS, Lloyd DB: **Modulation of firefly luciferase stability and impact on studies of gene regulation**. *Gene* 1991, **103**(2):171-177.

22. Ignowski JM, Schaffer DV: **Kinetic analysis and modeling of firefly luciferase as a quantitative reporter gene in live mammalian cells**. *Biotechnol Bioeng* 2004, **86**(7):827-834.

23. Corish P, Tyler-Smith C: **Attenuation of green fluorescent protein half-life in mammalian cells**. *Protein Eng* 1999, **12**(12):1035-1040.

24. Li X, Zhao X, Fang Y, Jiang X, Duong T, Fan C, Huang CC, Kain SR: **Generation of destabilized green fluorescent protein as a transcription reporter**. *J Biol Chem* 1998, **273**(52):34970-34975.

25. Niswender KD, Blackman SM, Rohde L, Magnuson MA, Piston DW: **Quantitative imaging of green fluorescent protein in cultured cells: comparison of microscopic techniques, use in fusion proteins and detection limits**. *J Microsc* 1995, **180 ( Pt 2)**:109-116.

26. Wendland M, Bumann D: **Optimization of GFP levels for analyzing Salmonella gene expression during an infection**. *FEBS Lett* 2002, **521**(1-3):105-108.
